# Anti-inflammatory potential of a malleable matrix composed of fermented whey proteins and lactic acid bacteria in an atopic dermatitis model

**DOI:** 10.1186/1476-9255-4-6

**Published:** 2007-03-21

**Authors:** Josée Beaulieu, Claude Dupont, Pierre Lemieux

**Affiliations:** 1 Institut national de la recherche scientifique, INRS-Institut Armand-Frappier, 531 boul. des Prairies, Laval, Québec, Canada, H7V 1B7; 2 Technologie Biolactis, 500 boul. Cartier suite 218, Laval, Québec, Canada, H7V 5B7

## Abstract

**Background:**

Over the last 10 years, whey proteins have received considerable attention in the area of functional foods and nutraceuticals. In this paper, a novel fermented whey protein-based product described as a gel-like Malleable Protein Matrix (**MPM**) has been tested for its anti-inflammatory activity. Preliminary *in vitro *results have already indicated that MPM could exert such an anti-inflammatory activity.

**Methods:**

The systemic anti-inflammatory activity of the MPM was explored using the oxazolone-induced atopic contact dermatitis mouse model (ACD). Parameters including ear thickness, side effects as well as neutrophil extravasation were monitored.

**Results:**

In the ACD model, the MPM exhibited an anti-inflammatory effect comparable to that of hydrocortisone (positive control). Mice fed with MPM showed strong reduction of the ear inflammation while no side effects, as compared to hydrocortisone, were observed. The MPM seemed to reduce neutrophil extravasation in tissue as evidenced by blood polymorphonuclear cells and ear myeloperoxidase content.

**Conclusion:**

The anti-inflammatory activity demonstrated in the ACD model suggests that the mechanism of action of the MPM is different than that of hydrocortisone and could become a relevant product for people suffering from dermatological manifestations associated with immune dysfunctions such as allergies, eczema, dermatitis, and autoimmune diseases.

## Background

Modern life-styles which leads to obesity, stress and inactivity, is a major cause of immunological diseases, particularly those associated with chronic inflammation which are on the upswing during the last decade [1-3]. Many evidences exist that functional foods have protective effects on immune deficiency [4-6] including whey proteins, which can modulate some immune functions [5]. Other studies revealed that whey proteins possess a myriad of activities including antioxidant activity attributed to increasing glutathione content [7,8], anti-allergic, [9] anti-inflammatory [9-11] and immunomodulatory activities [12-19]. Whey proteins such as β-lactoglobulin (β-LG), bovine serum albumin (BSA) and α-lactalbumin (α-LA) have been shown to stimulate splenocyte proliferation, increase interleukin-1 production by macrophages and increase GSH production [18,19]. Whey peptides have recently been shown to possess immunomodulatory activities such as a stimulation of lymphocytes, an increasing in phagocytosis process as well as in secretion of immunoglobulin A (IgA) by Peyer's patches [5,13,17,20].

Lactoferrin (LF), a minor whey protein, has been extensively studied. LF assists the phagocytosis process in neutrophils, increases production of interleukin-8 (IL-8) [13] and stimulates immune cell production [15,19,21]. Moreover, LF has also demonstrated anti-inflammatory effects in animal models by an inhibition of pro-Th1 cytokines and an increasing in regulatory cytokine IL-10 production [9,11]. More specifically, LF exerts its anti-inflammatory effect during mouse atopic contact dermatitis (ACD) by reducing ear thickness and infiltration of inflammatory cells following a direct topical contact [11].

In addition, some Lactic Acid Bacteria (LAB) have shown immunomodulatory and anti-inflammatory activities. The genus *Lactobacillus *commonly used in many fermented dairy products [22] is the most studied of these probiotics [23]. The effects of LAB are very strain-dependent but many *lactobacilli *act on Peyer's patches to stimulate IgA production, phagocytosis process and possess anti-inflammatory and anti-allergic activities by reducing the production of cytokines and immunoglobulin E (IgE) [24-27]. Cytokine production is also strain-dependent as some *lactobacilli *are able to increase Th1 profile while others increase Th2 profile [28]. These results suggest that *lactobacilli *could act both as immunostimulating and anti-inflammatory agents. Some studies also indicate that the effects of probiotics acting in synergy with food ingredients can be more intense than the probiotics alone [29]. Moreover, vitamins present in the MPM (niacin and riboflavin) as well as calcium also possess immunomodulatory effects [30-32].

Considering the positive effects on the immune system of both whey proteins and probiotic *lactobacilli*, a novel fermented whey protein-based ingredient, called Malleable Protein Matrix (MPM) [33], was tested for its immunomodulatory activities [34]. It was previously demonstrated that MPM stimulates production of blood polymorphonuclear cells, cytokine IL-18 as well as glutathione by white blood cells in healthy rat suggesting a stimulation of innate immunity [33,34]. On the other hand, MPM can also reduce the production of important pro-inflammatory cytokines such as TNFα [33]. Moreover, it was shown *in vitro *that MPM reduces pro-inflammatory cytokines and inhibits the cytokines production following LPS stimulation on CaCo2 cells [33]. These results suggested that MPM might also exhibit anti-inflammatory properties when placed in the context of inflammation.

The objective of this present study was to evaluate the systemic anti-inflammatory potential of MPM and to determine how its complex composition may lead to synergistic effects. For this purpose, the oxazolone-induced atopic contact dermatitis mouse model (ACD) was used. This ACD mouse model requires two distinct phases [35]. First, the sensitization phase is initiated by topical application of oxazolone, which permits the activation of T cells through Langerhans cells acting as an antigen presenting cells. The elicitation phase is next achieved by a subsequent topical application of oxazolone, which initiate the inflammatory process by recruiting activated T effector cells which in turn attract inflammatory cells [36-38]. The inflammatory cells recruited in this ACD model are principally macrophages, which attract neutrophils in the early inflammatory phase and monocytes as well as dendritic cells in the early and late inflammatory phases. CD4+ T cells act as regulatory cells and not as effector cells in the ACD model, in which they control the intensity of inflammatory reaction [39,40]. A similar dermatitis model has recently been used to evaluate the anti-inflammatory activity of LF [11] and a milk-product fermented by *Lactobacillus casei *[27].

## Methods

### Reagents

The Malleable Protein Matrix (MPM) was obtained from Technologie Biolactis inc. *(LaBaie, Qc, Canada)*. Briefly, the MPM is obtained by a protein specific recuperation procedure following the fermentation of sweet whey by a proprietary *Lactobacillus kefiranofaciens *strain (R2C2) isolated from kefir grains and adapted to grow in whey [33]. The composition of MPM is shown in Table 1. On a humid basis (w/w), the MPM contains 80% water, 8% protein, 6% minerals (2% calcium), 5% carbohydrate (2.7% lactose) and less than 1% of fat. Lyophilized MPM required reconstitution in water: 20 g of lyophilized MPM was blended with 80 mL of water for 2 minutes at maximum speed (20% w/v). The final reconstituted product is stable at 4°C for at least 1 month. Water-soluble hydrocortisone (HC) was obtained form Sigma-Aldrich Canada *(Oakville, On, Canada) *and was diluted in deionized double-distilled water to a final concentration of 10 mg/mL. For the mouse ACD model, the 4-ethoxy-methylene-2-phenyloxazol-5-one (oxazolone) *(Sigma-Aldrich Canada) *was required at a concentration of 5% (w/v) in acetone to cause inflammation.

**Table 1:** Composition of MPM

	***Composition (g/100 g)***
Protein	8.1
Lipids	0.9
Ash (minerals)	5.1
Carbohydrates	4.6
Lactose	2.7
Galactose	0.2

	***Minerals (mg/100 g)***

Potassium	142.9
Sodium	175.2
Calcium	1600
Phosphorus	730.3
Selenium	< 0.1
Magnesium	5.4

	***Oligo-elements (mg/100 g)***

Copper	0.07
iron	0.24
Manganese	0.05
Zinc	0.13

	***Vitamins (mg or μg/100 g)***

Riboflavine (B2)	0.32 mg
Niacin (B3)	1.00 mg
Pyridoxine (B6)	0.04 mg
Cobalamine (B12)	Not detected
Ascorbic acid (C)	Not detected
Folic acid	5 μg

	***Bacterial count (CFU/100 g)***

LAB	6 × 10^11^

### Animals

CD-1 female mice were obtained from Charles River Laboratories *(St-Constant, Qc, Canada) *and were used at 20 days of age for studies in the mouse ACD model. The animals were housed in filter top isolator cages in a room kept at 20–23°C with humidity maintained between 35–45% with a 12-hour light-dark cycle and free access to a standard laboratory pelleted Rodent Lab Diet 5001 *(Ren's Feed & Supplies Limited, Oakville, On)*. The experimental protocols used were approved by the Animal Care Committee of the INRS- Institut Armand-Frappier (Comité Institutionnel des Soins aux Animaux et de leur Utilisation (CISAU)) and were performed in accordance with the recommendations of the Canadian Council on Animal Care as specified in the Guide to the Care and Use of Experimental Animals (CISAU # 0306-01 and # 0410-01).

### Mouse atopic contact dermatitis (ACD)

After a week adaptation in the animal facility, the mice were separated in groups of 10 animals. The grouping was randomized according to the weight of the rodents. The murine model of ACD was based on those firstly described by Garrigue et *al*. [41] and modified as follows: abdomen hair of CD-1 mice was removed and the sensitization phase was done by the application of 100 μL of oxazolone 5 % (w/v) in acetone on the hairless abdomen. After four days, the elicitation phase (first challenge) was initiated by the application of 50 μL of oxazolone 5% (w/v) in acetone on the right ear (25 μL each side of the ear). The second challenge was done 7 days after the first challenge with the same procedure. The ear thickness of the mice was measured every day with a digital caliper *(VWR, Mont-Royal, Canada)*.

### Dose-response curve

The dose-response curve has been done in the prophylactic anti-inflammatory mouse ACD model. Groups of 10 CD-1 mice received each day by gavages (per os (p.o)), 100 μL of reconstituted lyophilized MPM at three doses 20% (w/v), 10% (w/v) and 5% (w/v), 100 μL of water or 100 μL of water-soluble hydrocortisone (10 mg/mL). The mouse ACD was performed as described previously and ear thickness was measured every day.

### Prophylactic protocol – Mouse ACD

The prophylactic anti-inflammatory potential of MPM was evaluated by the administration of MPM seven days prior to sensitization. Groups of 10 CD-1 mice received each day by gavages (per os (p.o)), 100 μL of reconstituted lyophilized MPM, 100 μL of water or 100 μL of water-soluble hydrocortisone (10 mg/mL). The mouse ACD was performed as described previously and ear thickness was measured every day. The mice's weight was measured twice a week. The spleen's weight was measured at the end of the protocol and was normalized in accordance to each mouse's weight.

### Therapeutic protocol – Mouse ACD

The therapeutic anti-inflammatory potential of MPM was evaluated by the administration of MPM, soluble hydrocortisone or water as in the prophylactic protocol, but only after the first challenge. The other parameters were followed as described.

### Evaluation of peripheral white blood cell counts

At the end of the prophylactic protocol of mouse ACD, the blood of each mouse was taken and white blood cell counts evaluated by flow cytometry. Briefly, the red blood cells were lysed with Optilyse C *(Beckman-Coulter, Fullerton) *in accordance with manufacturer's instructions. The cell counts were obtained by passage of 20 μL of preparation in a Flow Cytometry Epics XL cytometer (*Beckman Coulter, Fullerton*). The lymphocytes, monocytes and polymorphonuclears (PMN) were separated in accordance with cell size and cell granulometry.

### Evaluation of ears-myeloperoxidase (MPO) content

The method for the evaluation of MPO content was adapted from those developed by Bradley et al. [42] and Xia and Zweier [43]. The mice were sacrificed at the end of prophylactic protocol by CO2 and the ears were immediately remove and frozen quickly in liquid nitrogen. The ears were chopped up and added in 50 mM phosphate potassium buffer, pH 6.0 supplemented with 0.5% hexadecyltrimethylammonium bromide (HTAB). The ears were disrupted with three cycles of sonication (10 sec.) in water-ice bath followed by three freeze-thaw cycles in methanol-dry ice bath and another three cycles of sonication in water-ice bath. The homogenates were centrifuged at 10 000 g for 15 min at 4°C and the supernatants were conserved at -80°C until analyses. For the quantification of MPO content, 100 μL of homogenates (or MPO standard from *Sigma-Aldrich, Oakville, On*) were mixed with 2.9 mL of 50 mM phosphate potassium buffer containing 0.117 mg/mL of o-dianisidine (*Sigma-Aldrich, Oakville, On*) and 0.0005% hydrogen peroxide. The oxydation of o-dianisidine kinetic was followed at 460 nm with a spectrophotometer Varian Cary 300 (*Varian, St-Laurent, QC*) during 5 min at 25°C.

### Statistical analysis

The inflammatory mouse ACD experiments were performed with groups counting 10 mice/group and two independent experiments. The statistical analysis of data was performed by the biostatistical service of INRS-Institut Armand-Frappier. Statistical analysis used was a repeated measure one-way ANOVA test that permits the comparison between groups during the entire experiment independently of each day. When the ANOVA test was not possible because of interactions between groups, a Student test was run for comparison of groups at each day.

## Results

**Figure 1:**
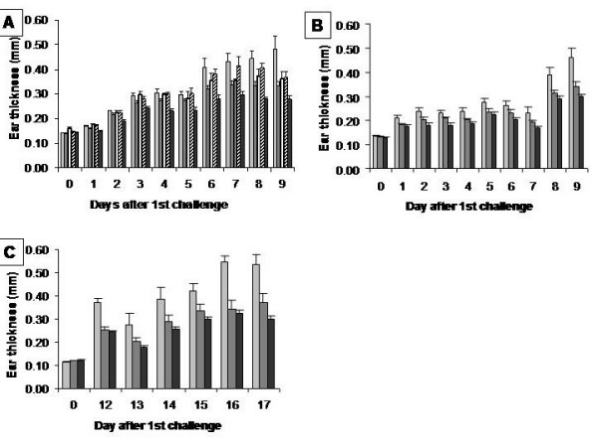
**Ear thickness of mice administered p.o. with the MPM, hydrocortisone or water**. A. Dose-response curve during the prophylactic model : Administrations started 7 days prior sensitization and challenges with oxazolone (p < 0.07 for MPM 20% and hydrocortisone groups compared with water reference group in the ANOVA statistical analysis). Legend: Light-grey bars: Water, Dark-grey bars: MPM 20%, White bars: MPM 10%, Hashed bars: MPM 5%, Black bars: Hydrocortisone. B. Prophylactic model: Administrations started 7 days prior sensitization and challenges with oxazolone (p < 0.07 for MPM and hydrocortisone groups compared with water reference group in the ANOVA statistical analysis) Legend: Light-grey bars: Water, Dark-grey bars: MPM, Black bars: Hydrocortisone. C. Therapeutic model: Administrations started after sensitization but during oxazolone challenges (p < 0.05 for MPM and hydrocortisone groups compared to water reference group in the ANOVA statistical analysis from day 8 until the end of experiment). Legend: Light-grey bars: Water, Dark-grey bars: MPM, Black bars: Hydrocortisone. (n = 10)

MPM is a whey-fermented product, which by its composition, has a high potential as an anti-inflammatory agent. The oxazolone-induced atopic contact dermatitis (ACD) model was used for the demonstration of MPM's effect on inflammatory diseases. Figure 1 shows an important reduction of ear thickness in mice consuming MPM as compared to that of the water control group. In the dose-response curve experiment, it is demonstrated that MPM possesses a higher anti-inflammatory effect when the concentration of product was 20% (Figure 1A). Consequently, MPM has been used for all experimentations at 20%. In the prophylactic protocol (Figure 1B), the maximal reduction of ear thickness was in the order of 26% in the MPM group and 35% in the hydrocortisone group as compared to the water control group. This thickness reduction was observed immediately after the first challenge and increases markedly after the second challenge. The ANOVA test indicates that reduction of ear thickness in the MPM group was statistically different of those from water group (p < 0.07) for the entire experiment. However, the ANOVA between MPM and HC groups was not possible because of interaction between the two groups. However, Student test has confirmed that the difference between both groups is not statistically different for the entire experiment (with exception for day 4). This statistical analysis permits to conclude that the anti-inflammatory effect of MPM is comparable to that of hydrocortisone treatment. In the therapeutic protocol (Figure 1C), the reduction of ear thickness was statistically different only after the second challenge in the MPM group compared to the water control group and reached a maximal reduction of 37%. For the group treated with hydrocortisone the maximal reduction of ear thickness reached 40%. Using the prophylactic protocol, these anti-inflammatory observations were confirmed in another independent experiment with the same batch of MPM and also with two other different batches of MPM. The results obtained were similar and statistically significant as confirmed by the ANOVA analysis, indicating the reproducibility of anti-inflammatory effect using different batches of MPM (data not shown).

**Figure 2:**
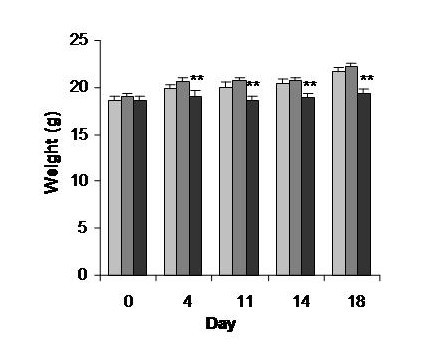
**Mice weight during the prophylactic ACD model**. Legend: Light-grey bars: Water, Dark-grey bars: MPM, Black bars: Hydrocortisone. (* p < 0.05) (n = 10)

**Figure 3:**
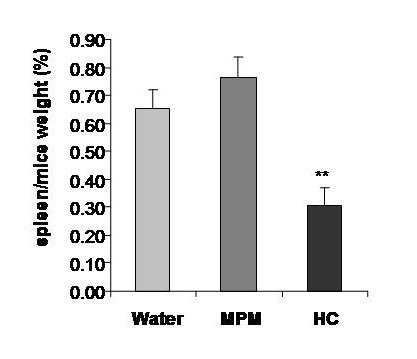
**Mice spleen weight at the sacrifice in the prophylactic ACD model**. Legend: Light-grey bars: Water, Dark-grey bars: MPM, Black bars: Hydrocortisone. (** p < 0.01) (n = 10)

**Figure 4:**
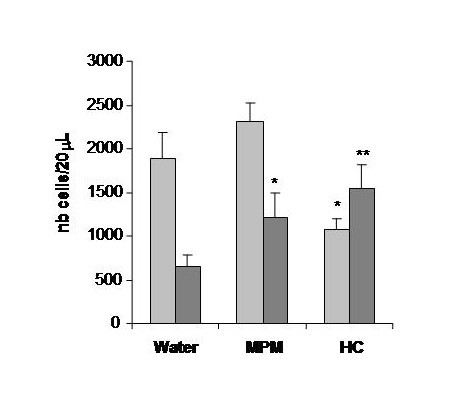
**Circulating cell counts 17 days after the first oxazolone challenge in the prophylactic ACD model**. Legend: Light-grey bars: Lymphocyte counts, Dark-grey bars: PMN counts (* p < 0.05; ** p < 0.01) (n = 10)

The consumption of hydrocortisone is associated with a negative effect on mice growth which is clearly demonstrated by the cessation of growth in the mice who received hydrocortisone (Figure 2). The MPM demonstrated an absence of detrimental effect on growth in comparison to water control group. Moreover, the hydrocortisone treatment induced a spleen atrophy represented by a 50% reduction in spleen weight as compared to water or MPM consumption (Figure 3). This spleen atrophy indicates an immunosuppression of immune cells after hydrocortisone treatment. No statistical difference was observed between the water and MPM group on spleen weight suggesting no immunosuppression following MPM consumption. The cell counts confirmed this immunosuppression following hydrocortisone treatment as demonstrated by the important reduction (approximately 50%) in circulating lymphocytes in comparison to water control group (Figure 4). On the contrary, the MPM consumption showed a tendency to increase lymphocyte numbers. These results indicated that MPM consumption do not induce side effects generally associated with hydrocortisone treatment.

**Figure 5:**
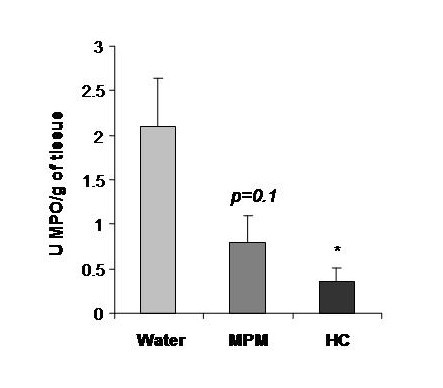
**Myeloperoxidase (MPO) contents in ears 18 days after the first oxazolone challenge during the prophylactic ACD model**. (* p < 0.05) (n = 10)

The polymorphonuclear (PMN) cell counts were higher in MPM and hydrocortisone fed groups compared to the water control group (Figure 4). The blood PMN counts were 1.86 and 2.35 fold higher in MPM and hydrocortisone respectively indicating a possible diminution of PMN extravasation in the ear of mice. The diminution of neutrophils extravasation as suggested by blood PMN counts was confirmed by the reduction of neutrophil content in ear of 62.4% and 82.6% following MPM and hydrocortisone treatment respectively, as measured by myeloperoxidase (MPO) ear analysis (Figure 5).

## Discussion

MPM contains a variety of ingredients including whey proteins and peptides, LAB and their related exopolysaccharides, group B vitamins and calcium (Table 1). All these ingredients possess effects on the immune system such as an interesting anti-inflammatory potential [5,14,15,32,44,45]. In light of these components, the MPM is believed to possess an anti-inflammatory potential, which may be amplified by the synergy of its individual components. Previous observations suggested that the MPM could be an interesting treatment in inflammatory diseases. Indeed, it was demonstrated that MPM reduced production of cytokine TNFα in healthy rat [33]. This cytokine is very important in the development of the ACD disease and contributes in the amplification of inflammatory reaction [46]. The reduction of this pro-inflammatory cytokine following MPM consumption indicated its potential in the inhibition of development of inflammatory disease and reduction of its intensity. Moreover, MPM inhibited the production of cytokines *in vitro *on CaCo2 cells stimulated with LPS [33] suggesting the inhibition of development of inflammation following an inflammatory stimulus.

The anti-inflammatory potential of MPM has been confirmed in these studies with the murine ACD model. This model of inflammation has proven to be a sensitive and useful tool to determine efficacy and potency of several anti-inflammatory and immunosuppressive drugs used in dermatological disorders such as dermatitis and psoriasis. Glucocorticoids, such as hydrocortisone, are commonly used to relieve skin and joint inflammation and have been used as a positive control group in these experiments [35]. This model comprises two important phases in order to examine inflammation: 1) Sensitization phase that is developed by application of oxazolone on the abdomen, allowing the recruitment of antigen presenting cells, which capture and present the antigen (oxazolone) to naive T lymphocytes that afterwards become active. 2) Elicitation phase developed by the application of oxazolone on the ear, which allows activation of T lymphocytes to move to the ear and recruit inflammatory cells [35,47].

MPM and hydrocortisone administered *p.o*. either in a prophylactic (Figure 1B) or a therapeutic fashion (Figure 1C) reduced the inflammation with similar efficiency as demonstrated by the reduction of ear redness and thickness. In the prophylactic protocol (Figure 1B), the reduction of ear inflammation was observed as soon as one day after the first challenge and this protective effect was conserved throughout the entire course of the experiment. On the other hand, for therapeutic protocol, the anti-inflammatory effect following MPM consumption was apparent only after the second challenge (Figure 1C). The effect of MPM in this model (therapeutic protocol) showed that a certain period of time is required to overcome existing inflammation. This indicates that the MPM possesses an anti-inflammatory effect in an existing disease and is not only a preventive treatment. This therapeutic effect is interesting because those who suffer from such disease can consume MPM during crisis and will benefit of its effect. This study has shown that the reduction of inflammation by MPM consumption is not negligible as demonstrated by the comparison with hydrocortisone treatment. From that observation, we could speculate that the MPM might also exerts a beneficial effect on the reduction of skin itching and pain.

The MPM has a strong anti-inflammatory effect as demonstrated by its ability to reduce dermatological inflammation to the same extent than that of hydrocortisone. However in contrast to hydrocortisone, the MPM showed no side effects generally associated to medication including spleen atrophy, reduction in lymphocyte circulating cells or deleterious effect on body weight gain (Figures 2, 3 and 4). Hydrocortisone exerts its anti-inflammatory potential by suppression of immune cells. The reduction of inflammation observed by hydrocortisone treatment corresponded to a suppression of total immune cells (not only those implicated in inflammation), which was seen by the reduction in blood lymphocytes (Figure 4) and in spleen weight (Figure 3) for the mice consuming hydrocortisone. Consequently, people treated by hydrocortisone will be in a general immunosuppressed state and are therefore, more susceptible to contract other diseases and infection. No reduction in immune cells or spleen atrophy was observed in the mice who consumed MPM in comparison with the control water group. In fact, a trend showing immune stimulation by the MPM consumption was observed as indicated by the tendency to increase lymphocytes counts as well as spleen weight.

Atopic dermatitis is a disease that affects young children consequently, the use of hydrocortisone would not be advisable because of its inhibitory properties on growth [48]. This inhibition in growth following hydrocortisone consumption has been demonstrated in this study where the growth of these young mice treated with hydrocortisone was stopped during all the experiment (Figure 2) in comparison with mice treated with MPM and water which gained weight. Consequently, consumption of MPM by children and young adult in replacement of hydrocortisone as an anti-inflammatory product would be a good alternative.

The absence of all these detrimental effects by MPM consumption suggests that the mechanism of its anti-inflammatory action is different than that of hydrocortisone. However, both hydrocortisone and MPM seem to inhibit neutrophil extravasation and accumulation in inflamed tissues as shown with a higher polymorphonuclear cells (PMN) in circulation as well as a reduced MPO content in ear (Figures 4 and 5). Results in figure 4 demonstrate an inverse correlation between inflammation and PMN counts where in the hydrocortisone and MPM groups, the blood PMN counts is higher while the ear thickness is lower than reference water group. These results are consistent with those observed for ear MPO content (Figure 5). The MPO is an enzyme exclusively present in neutrophil granules and its enzymatic activity measured in a tissue is in direct correlation of the levels of neutrophils in a tissue [42]. The MPO results showed that the neutrophil infiltration in ear of mice that received hydrocortisone and MPM is reduced compared to the mice receiving water. The blood PMN count parameter and ear MPO content could be explained by the fact that in ACD, the neutrophils (the most important group in PMN) move from blood to ear because these cells are principally responsible for inflammation [36-38]. The hydrocortisone as well as the MPM seems to prevent the neutrophil extravasation from blood to ear, reducing the ear inflammation. However, the mechanism causing this inhibition of neutrophil extravasation is different between these two groups because of the absence of immunosuppression in MPM group as seen by the absence of spleen atrophy as well as blood lymphocyte counts (Figures 3 and 4). This inhibition of neutrophil infiltration indicate that MPM will be a good candidate for the treatment or prevention of neutrophilic diseases such as, Sweet syndrome (a neutrophilic dermatose resulting of Crohn's disease complications) as well as chronic obstructive pulmonary disease [49,50].

It is previously demonstrated that MPM enhances some cytokines, blood PMN cells and glutathione production by leukocytes [33] indicating that MPM exerts a definitive immunomodulation. Its consumption could either be beneficial in a context of stimulation of innate immunity but detrimental in the context of inflammatory disease. This present study reveals the interesting properties of MPM in the reduction of inflammation confirming that despites its innate immunity stimulation potential, MPM act also as an anti-inflammatory agent. The complexity of MPM components as well as the potential synergy between its components could explain the properties of MPM to be an immunomodulatory agent as well as to be an anti-inflammatory agent in the context of inflammation. These two different immune situations suggest that MPM act trough a regulatory mechanism explaining their both immunomodulatory and anti-inflammatory properties. These results demonstrate that, as a new product, the Malleable Protein Matrix reduces inflammation and immune dysfunctions when consumed orally while maintaining an appropriate immune system threshold. Experiments to demonstrate the mechanism of action responsible for the anti-inflammatory effect of the MPM consumption and other parameters to determine how specific cells are implicated and influenced by MPM consumption in this ACD model are underway.

## Conclusion

MPM possesses a strong anti-inflammatory effect comparable to hydrocortisone when examined in the ACD model. The anti-inflammatory effects of consumption of MPM occur without the undesirable side effects normally associated with hydrocortisone. Therefore, MPM would be an alternative of choice for children and young adult suffering from chronic inflammatory of various diseases such as ACD. The consumption of the MPM could act as a preventive or a therapeutic nutraceutical in the case of inflammatory diseases like atopic dermatitis or related diseases such as, psoriasis. Psoriasis is a chronic inflammatory disease with similar effects on the immune system to that observed for ACD.

## Competing interests

Technologie Biolactis (TB) was the industrial sponsor of a Natural Science and Engineering Research Council of Canada (NSERC) grant obtained by INRS (CD). Collaborative research conventions and agreements intervened between TB, INRS and NSERC. INRS is a minor shareholder of TB (less than 1%) and does not have any vote. The findings of the present study are covered by a patent application (PCT CA2002/001899). JB was an on-site scholar of Fond de Recherche en Santé du Québec (FRSQ) and part of the scholarship was covered by TB.

## Authors' contributions

JB design the animal studies, carried out the animal and other experiments, perform the statistical analysis and drafted the manuscript. CD participated in the design of animal studies, data interpretation and the statistical analysis. CD revised the manuscript for the intellectual content and language. PL participated in the design of animal studies, data interpretation and revised the manuscript for the intellectual content and language. All authors read and approved the final manuscript.
